# Association of advanced glycation end products with ear lobe crease: A cross‐sectional study

**DOI:** 10.1111/1753-0407.13548

**Published:** 2024-04-10

**Authors:** Yuepeng Wang, Yu Xin, Binqi Li, Qingzheng Wu, Ping An, Bing Li, Yijun Li, Li Zang, Weijun Gu, Yiming Mu

**Affiliations:** ^1^ Department of Endocrinology The First Medical Center of Chinese PLA General Hospital Beijing China; ^2^ School of Medicine Nankai University Tianjin China

**Keywords:** advanced glycation end products, cardiovascular disease, ear lobe crease, type 2 diabetes

## Abstract

**Objective:**

Several studies have demonstrated a significant association between the presence of the ear lobe crease (ELC) and cardiovascular disease. Advanced glycation end‐products (AGEs) can affect the structures and functions of proteins and contribute to the development of diabetic complications. However, few studies have reported the relationship between AGEs and ELC. The purpose of this study was to investigate the correlation of skin autofluorescence (SAF)‐AGE_age_ (SAF‐AGEs × age/100) with ELC.

**Methods:**

This cross‐sectional study enrolled 6500 eligible participants from two communities in Beijing. Skin autofluorescence (SAF) was used to measure skin AGEs (SAF‐AGEs). SAF‐AGE_age_ was defined as AGEs × age/100. Binary logistic regression analysis and linear regression analysis nested in logistic models were applied to test outcomes.

**Results:**

The overall prevalence of ELC with an average age of 62.7 years participants was 57.1% (*n* = 3714). Age, fasting blood glucose, systolic blood pressure, and lipoprotein cholesterol were all greater in participants with ELC. ELC‐positive participants had higher prevalence of coronary heart disease. Logistic analysis showed a significantly positive relationship between quartiles of SAF‐AGE_age_ and ELC (odds ratio [OR] 1.526, 95% CI 1.324–1.759; OR 2.072, CI 1.791–2.396; and OR 2.983, CI 2.551–3.489) for the multivariate‐adjusted models, respectively. Stratified research revealed that those with a history of diabetes, hypertension, or coronary heart disease experienced the connection between SAF‐AGE_age_ and ELC.

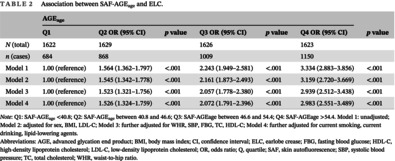

**Conclusion:**

ELC is associated with coronary heart disease, and the SAF‐AGE has a potential role in ELC development in elder people.

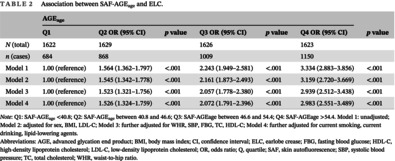

## Graphical abstract

Highlights

- Patients with ear lobe crease (ELC) are more likely to have carotid artery lesions stroke and coronary heart disease.
- Skin autofluorescence advanced glycation end products (SAF‐AGE_age_) was associated with the prevalence of ELC.

## 1 INTRODUCTION

**FIGURE 1:**
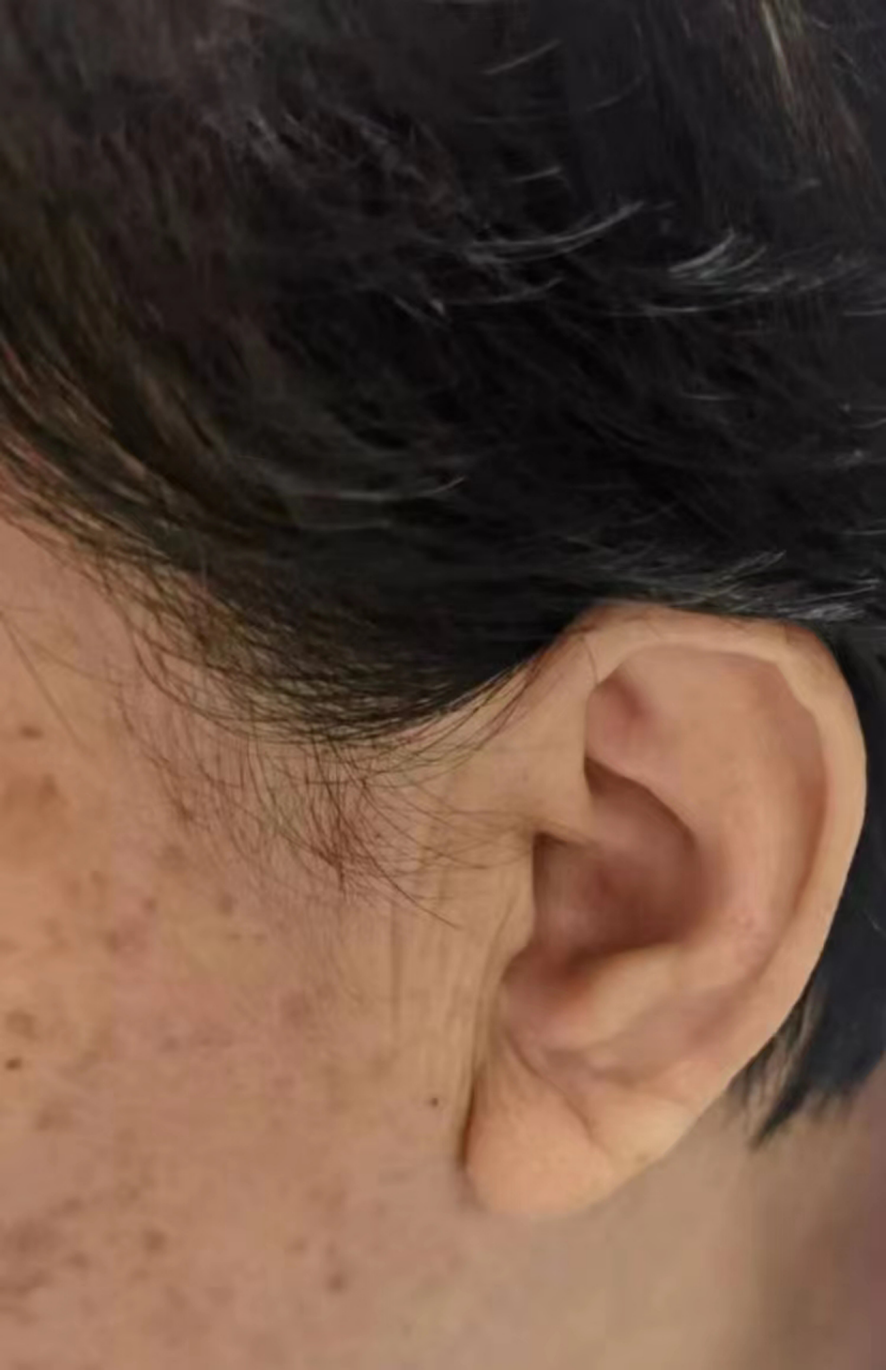
Schematic of patient's ear lobe crease. A 61‐year‐old woman with a background of diabetes mellitus, hypertension, and hyperlipidemia was observed with earlobe crease. Angiography revealed severe triple vessel disease.

Multiple studies have demonstrated a significant correlation between the presence of ear lobe crease (ELC) and the occurrence of coronary heart disease (CHD). ELC refers to a diagonal earlobe crease that extends at a 45° angle backwards from the tragus to the auricle (Figure 1), and it is postulated to serve as a potential indicator of atherosclerotic disease. The prevalence of diagonal ELC has been observed to rise with advancing age and the severity of CHD and other atherosclerosis‐related diseases.^1^, ^2^, ^3^ Furthermore, the presence of a deep ELC was independently associated with the increased risk of adverse outcome in acute ischemic stroke.^4^ A comprehensive meta‐analysis has further confirmed the association between ELC and coronary artery disease.^5^ Stoyanov et al have provided evidence of the correlation between cardiac and ELC samples, exhibiting characteristics of arterial myoelastofibrosis and tissue fibrosis by biopsies.^6^ Nevertheless, the etiology of ELC remains uncertain, although it appears to be associated with the depletion of elastin and elastic fibers.^7^ Previous studies have proposed potential mechanisms for ELC, including skin aging, collagen degeneration, and telomere shortening.^8^

Diabetes is widely recognized as a prominent risk factor for cardiovascular disease. The increased susceptibility to cardiovascular disease in individuals with diabetes primarily stems from the detrimental effects of hyperglycemia, tissue‐specific insulin resistance, and chronic inflammatory responses induced by reactive oxygen species and advanced glycation end products (AGEs).^9^ AGEs, which are diverse compounds formed through nonenzymatic glycation of amino groups on proteins via Maillard reactions, accumulate more prominently in a state of persistent hyperglycemia. The formation of endogenous AGEs in diabetes leads to tissue damage by damaging protein structures and functions, particularly collagen.^10^ It has been reported that AGEs accumulate in extrinsically aged skin at high levels.^11^ Taking advantage of the characteristic autofluorescent properties of AGEs, skin autofluorescence (SAF) offers an excellent opportunity for non‐invasive investigation of glycation with reliable accuracy.^10^ SAF‐AGE has been proposed as a prognostic factor for developing diabetic complications.^12^ Ying et al have reported the correlation between age‐combined AGEs, that is, AGE_age_ (defined as AGEs × age/100) for carotid atherosclerotic disease and lower limb atherosclerosis in patients with type 2 diabetes mellitus (T2DM).^13^, ^14^ AGEs also appear to be highly accumulated in extrinsically aged skin^11^ and the accumulation of SAF‐AGEs contributes to the loss of skin elasticity.^15^ Given the role of ELC in the prediction of cardiovascular disease in patients and the mechanism of AGEs in the development of cardiovascular disease and skin degeneration, we hypothesized that there might be a relationship between them. However, the relationship between ELC and AGEs has rarely been reported before. In view of the importance of age on AGEs and ELC,^16^ We used the age‐adjusted combined SAF‐AGE_age_ (SAF‐AGEs × age/100) to investigate the correlation between ELC and AGEs in the cross‐sectional study.

## 2 METHODS

### 2.1 Study population

The study was part of within the REACTION (Risk Evaluation of Cancers in Chinese Diabetic Individuals) study, a multisite prospective cohort study investigating the association ofT2DM with the risk of cancer in the People's Republic of China.^17^ From September 2018 to December 2018 6854 participants from two communities of Beijing (Laoshan and Gucheng communities) were included. Community‐based primary health care center staff assisted with subject recruitment. The inclusion criteria of our study were all participants for whom the ELC and the SAF measurement of AGEs were completed. Participants having history of cancer, history of end‐stage renal disease, or data missing were excluded from the study. Based on the criteria, the study ultimately enrolled 6500 eligible participants.

The study underwent review and received approval from the Institutional Research Ethics Committee of Rui Jin Hospital, affiliated with Shanghai Jiao Tong University School of Medicine (No. 2011‐14). The participants provided their written informed consent to participate in this study.

### 2.2 Clinical and laboratory measurement

Basic information about the participants was obtained by trained investigators using a standardized questionnaire, which covered the content of age, sex, medical history, familial medical history, medication history, smoking status, drinking status, and so forth. Coronary heart disease is self‐reported and includes asymptomatic and ischemic CHD and angina pectoris.

We measured height, weight, waist circumference, hip circumference, and blood pressure. After each subject had fully rested, blood pressure was measured three times in a sitting position using an electronic device, with an interval of 1 min between each measurement, and the average value was used in the analysis. Subjects with unilateral or bilateral oblique extension from the external auditory canal to the edge of the earlobe without discontinuity were considered ELC positive. Laboratory tests include alanine transferase, aspartate transferase (AST), serum total cholesterol (TC), triglycerides (TG), high‐density lipoprotein cholesterol (HDL‐C), low‐density lipoprotein cholesterol Lipoprotein cholesterol (LDL‐C), and fasting blood glucose (FBG). A 75 g oral glucose tolerance test (OGTT) was performed after collecting the fasting blood sample, and 2‐h postprandial blood glucose (2‐hPBG) was measured. For plasma glucose (including FBG and 2‐hPBG), blood samples were collected in tubes containing sodium fluoride and measured using the hexokinase method. Glycated hemoglobin (HbA1c) was determined using high‐performance liquid chromatography (VARIANT II system, Bio‐Rad, Hercules, CA). TC, TG, HDL‐C, and LDL‐C were measured using an autoanalyzer (ARCHITECT c16000 System; Abbott Laboratories, Chicago, IL).

Skin AGEs were measured using a spectrometer (AGE Reader, Hefei Institute of Physical Sciences, Chinese Academy of Sciences). Skin AGEs were measured as described in our previous study.^18^ Briefly, an excitation light source with a peak wavelength of 370 nm was used to illuminate a 1–4 cm section of the forearm and participants were instructed to remain still for the duration of the acquisition time (approximately 30 s). The SAF was calculated from the ratio of emitted and reflected light measured by the AGE reader. A total of three measurements were taken and averaged for analysis. SAF‐AGE_age_ was calculated as SAF‐AGEs × age/100.

### 2.3 Definition of variables

Hypertension is defined as systolic blood pressure (SBP) >140 mmHg and/or diastolic blood pressure (DBP) >90 mmHg or a self‐reported history of hypertension. T2DM is defined as HbA1c >6.5%, FBG >7.0 mmol/L, 2‐hPBG >11.1 mmol/L, or self‐reported history of T2DM.^19^ The criteria defining prediabetes were as follows: FBG of 5.6–6.9 mmol/L as impaired fasting glucose, 2‐hPBG of 7.8–11.0 mmol/L during OGTT as impaired glucose tolerance, or HbA1c of 5.7%–6.4%. Hyperlipidemia was defined as TC >5.17 mmol/L, TG >2.3 mmol/L or a self‐reported history of hyperlipidemia.

### 2.4 Statistical analysis

IBM SPSS statistics (version 26.0; SPSS, Inc, Chicago, IL) software was used to perform all analyses. Continuous variables are articulated as the mean ± SD or median, and categorical variables are expressed as frequency and percentage. The interquartile range was calculated according to a normal or skewed distribution. Continuous variables were compared using the one‐way analysis or nonparametric rank‐sum test for normal continuous variables, and categorical variables were compared with the *χ* ^2^‐test. In all models, covariates were selected based on the clinical relevance and univariate variable with interest. Independent variables were examined for multicollinearity. The test indicated that there was no multicollinearity (variance inflation factor <2). Binary logistic regression analysis and linear regression analysis were nested in logistic models to evaluate the relationships between the prevalence of ELC and quartiles of SAF‐AGE_age_. Statistical significance was determined by *p* < .05.

## 3 RESULTS

### 3.1 Clinical characteristics of the study population

**FIGURE 2:**
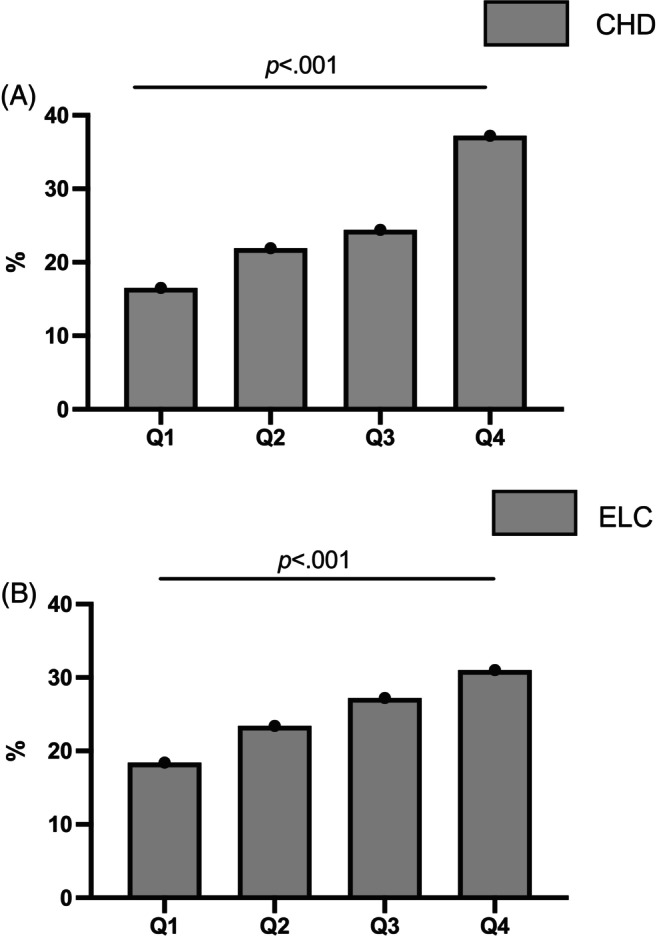
The prevalence of CHD (**A**) and ELC (**B**) in different tertiles of SAF‐AGE_age_ levels in middle‐aged or elderly Chinese participants. AGE, advanced glycation end product; CHD, coronary heart disease; ELC, earlobe crease; Q, quartile; SAF, skin autofluorescence.

Table 1 summarizes the clinical characteristics of the 6500 enrolled participants. The average age of participants in the study was 62.7 ± 7.8 years. The overall prevalence of ELC in these elderly participants was 57.1% (*n* = 3714). Mean SAF‐AGE_age_ in groups with and without ELC was 50.6 ± 11.3 and 45.6 ± 9.8, respectively (*p* < .001). Patients with ELC were significantly more likely to be male and older and to have higher SBP, FBG, and LDL‐C (all *p* < .05). In addition, the prevalence of ELC was significantly higher in patients who currently smoked, drank, and had diabetes. Patients with ELC are more likely to have CHD, stroke, and carotid artery lesions (all *p* < .05). With the increase of SAF‐AGE_age_ level, the prevalence of both CHD and ELC increased (Figure 2).

**TABLE 1:** Distribution of risk factors in relation to ELC.

	Total	Without ELC	With ELC	*p* value
*n*	6500	2789	3711	
Age, (years)	62.7 ± 7.8	60 ± 7.2	64.7 ± 7.6	<.001
Gender (male), *n* (%)	2231 (34.3)	779 (27.9)	1452 (39.1)	<.001
Menopause (only female), *n* (%)	4008 (94.9)	1820 (91.5)	2188 (98.0)	<.001
BMI, kg/m^2^	25.3 ± 3.3	25.2 ± 3.5	25.4 ± 3.3	.012
WHR	0.9 ± 0.1	0.9 ± 0.1	0.9 ± 0.1	<.001
SBP, mm Hg	133.1 ± 16.8	131.2 ± 16.6	134.6 ± 16.7	<.001
DBP, mm Hg	79 ± 9.6	79.2 ± 9.6	78.8 ± 9.6	.058
FBG, mmol/L	6.2 ± 1.9	6.1 ± 1.8	6.3 ± 2.0	<.001
2‐h PG, mmol/L	7 ± 4.6	7.1 ± 4.4	7.0 ± 4.7	.935
ALT, U/L	22.2 ± 18.2	22.6 ± 15.7	22 ± 20	.190
AST, U/L	24.2 ± 11.7	24.1 ± 10.5	24.2 ± 12.6	.574
TC, mmol/L	5.3 ± 1.0	5.4 ± 1.0	5.3 ± 1.0	<.001
TGs, mmol/L	1.7 ± 1.2	1.7 ± 1.3	1.6 ± 1.2	.367
HDL‐C, mmol/L	1.5 ± 0.4	1.5 ± 0.4	1.5 ± 0.3	<.001
LDL‐C, mmol/L	5.5 ± 3.8	5.2 ± 3.4	5.8 ± 4.0	<.001
SAF‐AGE_age_	48.5 ± 11.0	45.6 ± 9.8	50.6 ± 11.3	<.001
Current smoking, *n* (%)	938 (14.4)	353 (12.7)	585 (15.8)	.001
Current drinking, *n* (%)	1544 (23.8)	603 (21.6)	941 (25.4)	<.001
Diabetes, *n* (%)	1798 (27.7)	664 (23.8)	1134 (30.6)	<.001
Prediabetes, *n* (%)	576 (8.9)	231 (8.3)	345 (9.3)	.001
Hypertension, *n* (%)	2494 (38.4)	921 (33.0)	1573 (42.4)	<.001
Hyperlipidemia, *n* (%)	2104 (32.3)	884 (31.6)	1220 (32.9)	.272
Carotid artery intima‐media thickening, *n* (%)	1178 (18.1)	473 (17.0)	705 (19.0)	<.001
Carotid artery plaque, *n* (%)	3165 (48.7)	1017 (36.5)	2148 (57.9)	<.001
CHD, *n* (%)	787 (12.1)	257 (9.2)	530 (14.3)	<.001
Atrial fibrillation, *n* (%)	86 (1.3)	29 (1.0)	57 (1.5)	.083
Stroke history, *n* (%)	310 (4.8)	105 (3.8)	205 (5.5)	.001
Hypertension family history, *n* (%)	4016 (61.8)	1727 (61.9)	2289 (61.7)	.844
Diabetes family history, *n* (%)	2068 (31.8)	883 (31.7)	1185 (31.9)	.816
Lipid‐lowering agents, *n* (%)	1289 (19.8)	494 (17.7)	795 (21.4)	<.001

*Note*: Data are expressed as mean with SD or *n* (%).

Abbreviations: AGE, advanced glycation end product; ALT, alanine transferase; AST, aspartate transferase; BMI, body mass index; CHD, coronary heart disease; DBP, diastolic blood pressure; ELC, earlobe crease; FBG, fasting blood glucose; HDL‐C, high‐density lipoprotein cholesterol; LDL‐C, low‐density lipoprotein cholesterol; PG, postload blood glucose; SAF, skin autofluorescence; SBP, systolic blood pressure; TC, total cholesterol; TG, triglyceride; WHR, waist‐to‐hip ratio.

### 3.2 Correlation between SAF‐AGE_age_ quartile and ELC

Multiple logistic regression analysis was done to investigate the connection between the SAF‐AGE_age_ quartile and ELC (Table 2). Patients with higher SAF‐AGE_age_ levels had a higher likelihood of having ELC compared to participants in the SAF‐AGE_age_ first quartile in Model 1 to Model 3 (*p* < .001). After further adjusting for gender, BMI (body mass index), LDL‐C, waist‐to‐hip ratio (WHR), SBP, FBG, TC, HDL‐C, smoking, drinking, and use of lipid‐lowering agents, the correlation for Model 4 remained significant (odds ratio [OR] Q2 vs Q1: 1.526 [95% confidence interval (CI), 1.324–1.759], Q3 vs Q1: 2.072 [1.791–2.396], Q4 vs Q1: 2.983 [2.551–3.489], *p* for trend <.001).

**TABLE 2:** Association between SAF‐AGE_age_ and ELC.

	AGE_age_						*p* value
	Q1	Q2 OR (95% CI)	*p* value	Q3 OR (95% CI)	*p* value	Q4 OR (95% CI)	
*N* (total)	1622	1629		1626		1623	
*n* (cases)	684	868		1009		1150	
Model 1	1.00 (reference)	1.564 (1.362–1.797)	<.001	2.243 (1.949–2.581)	<.001	3.334 (2.883–3.856)	<.001
Model 2	1.00 (reference)	1.545 (1.342–1.778)	<.001	2.161 (1.873–2.493)	<.001	3.159 (2.720–3.669)	<.001
Model 3	1.00 (reference)	1.523 (1.321–1.756)	<.001	2.057 (1.778–2.380)	<.001	2.939 (2.512–3.438)	<.001
Model 4	1.00 (reference)	1.526 (1.324–1.759)	<.001	2.072 (1.791–2.396)	<.001	2.983 (2.551–3.489)	<.001

*Note*: Q1: SAF‐AGE_age_ <40.8; Q2: SAF‐AGE_age_ between 40.8 and 46.6; Q3: SAF‐AGEage between 46.6 and 54.4; Q4: SAF‐AGEage >54.4. Model 1: unadjusted; Model 2: adjusted for sex, BMI, LDL‐C; Model 3: further adjusted for WHR, SBP, FBG, TC, HDL‐C; Model 4: further adjusted for current smoking, current drinking, lipid‐lowering agents.

Abbreviations: AGE, advanced glycation end product; BMI, body mass index; CI, confidence interval; ELC, earlobe crease; FBG, fasting blood glucose; HDL‐C, high‐density lipoprotein cholesterol; LDL‐C, low‐density lipoprotein cholesterol; OR, odds ratio; Q, quartile; SAF, skin autofluorescence; SBP, systolic blood pressure; TC, total cholesterol; WHR, waist‐to‐hip ratio.

### 3.3 The relationship between SAF‐AGE_age_ and ELC in stratified analysis

After controlling for sex, hypertension, DM, drinking and smoking habits, BMI, WHR, SBP, FBG, TC, HDL‐C, and LDL‐C, stratified analysis was employed to further confirm the stability of the association between SAF‐AGEage and ELC in multiple populations (Table 3). Stratified by sex, SAF‐AGE_age_ in the fourth quartile was associated with increased ratio of ELC in women (OR [95% CI] Q2 vs Q1: 1.628 [1.377–1.924]; Q3 vs. Q1: 2.055 [1.726–2.447]; Q4 vs Q1: 3.051 [2.499–3.724], *p* trend <.001) and in men (OR [95% CI] Q3 vs Q1: 1.859 [1.411–2.451]; Q4 vs Q1: 2.443 [1.855–3.218], *p* trend <.001). The same trend was observed at the prediabetes group (OR [95% CI] Q3 vs Q1: 1.907 [1.172–3.104]; Q4 vs Q1: 2.502 [1.455–4.304], *p* trend = .007) and diabetes group (OR [95% CI] Q3 vs Q1: 1.814 [1.325–2.482]; Q4 vs Q1: 2.485 [1.809–3.412], *p* trend <.001). In subgroups of hypertension, nonhypertension, current smoking and noncurrent smoking, CHD and non‐CHD, compared with subjects in the lowest SAF‐AGE_age_ quartile, ORs [95% CI] for being with ELC in the highest SAF‐AGE_age_ quartile were 2.690 [2.084–3.472], 2.963 [2.413–3.639], 1.835 [1.218–2.763], 3.204 [2.699–3.803], 2.529 [1.520–4.207], and 2.744[2.341–3.287], respectively (all *p* trend <.001).

**TABLE 3:** Stratified analyses of the association of SAF‐AGE_age_ and ELC.

	AGE_age_				*p* value
	Q1	Q2	Q3	Q4	
Total					
No. of patients	1622	1629	1626	1623	
No. of cases	684	868	1009	1150	
Multivariable‐adjusted OR	1	1.518 (1.316–1.751)	2.030 (1.754–2.350)	2.871 (2.451–3.364)	<.001
Male					
No. of patients	381	485	577	788	
No. of cases	198	278	394	582	
Multivariable‐adjusted OR	1	1.238 (0.939–1.631)	1.859 (1.411–2.451)	2.443 (1.855–3.218)	<.001
Female					
No. of patients	1241	1144	1049	835	
No. of cases	486	590	615	568	
Multivariable‐adjusted OR	1	1.628 (1.377–1.924)	2.055 (1.726–2.447)	3.051 (2.499–3.724)	<.001
No diabetes					
No. of patients	1204	1098	968	856	
No. of cases	482	581	578	591	
Multivariable‐adjusted OR	1	1.648 (1.390–1.955)	2.094 (1.751–2.505)	3.065 (2.508–3.744)	<.001
Prediabetes					
No. of patients	140	133	157	146	
No. of cases	67	74	100	104	
Multivariable‐adjusted OR	1	1.525 (0.932–2.496)	1.907 (1.172–3.104)	2.502 (1.455–4.304)	.007
Diabetes					
No. of patients	278	398	501	621	
No. of cases	135	213	331	455	
Multivariable‐adjusted OR	1	1.168 (0.851–1.604)	1.814 (1.325–2.482)	2.485 (1.809–3.412)	<.001
With hypertension					
No. of patients	484	559	655	796	
No. of cases	237	325	430	581	
Multivariable‐adjusted OR	1	1.437 (1.118–1.849)	1.871 (1.458–2.402)	2.690 (2.084–3.472)	<.001
Without hypertension					
No. of patients	1138	1070	971	827	
No. of cases	447	543	579	569	
Multivariable‐adjusted OR	1	1.551 (1.304–1.846)	2.105 (1.754–2.525)	2.963 (2.413–3.639)	<.001
Current smoking (Yes)					
No. of patients	171	222	253	292	
No. of cases	86	128	174	197	
Multivariable‐adjusted OR	1	1.295 (0.895–1.951)	1.954 (1.290–2.96)	1.835 (1.218–2.763)	.004
Current smoking (No)					
No. of patients	1451	1407	1373	1331	
No. of cases	598	740	835	953	
Multivariable‐adjusted OR	1	1.547 (1.330–1.801)	2.062 (1.765–2.410)	3.204 (2.699–3.803)	<.001
With CHD					
No. of patients	95	158	218	316	
No. of cases	52	87	149	242	
Multivariable‐adjusted OR	1	0.956 (0.566–1.613)	1.675 (1.004–2.793)	2.529 (1.520–4.207)	<.001
Without CHD					
No. of patients	1527	1471	1408	1307	
No. of cases	632	781	860	908	
Multivariable‐adjusted OR	1	1.566 (1.350–1.818)	2.021 (1.733–2.358)	2.744 (2.341–3.287)	<.001

*Note*: Q1: SAF‐AGE_age_ below 40.8; Q2: SAF‐AGE_age_ between 40.8 and 46.6; Q3: SAF‐AGE_age_ between 46.6 and 54.4; Q4: SAF‐AGE_age_ above 54.4. Adjusted for sex, hypertension, diabetes, current smoking, current drinking, lipid‐lowering agents, BMI, WHR, SBP, FBG, TC, HDL‐C, LDL‐C.

Abbreviations: AGE, advanced glycation end product; BMI, body mass index; CHD, coronary heart disease; ELC, earlobe crease; CI, confidence interval; FBG, fasting blood glucose; HDL‐C, high‐density lipoprotein cholesterol; LDL‐C, low‐density lipoprotein cholesterol; OR, odds ratio; Q, quartile; SAF, skin autofluorescence; SBP, systolic blood pressure; TC, total cholesterol; WHR, waist‐to‐hip ratio.

## 4 DISCUSSION

This study investigated the relationship of SAF‐AGEs with ELC in the Chinese population and has the following main findings. ELC is associated with CHD, and with the categories of increasing SAF‐AGE_age_, the prevalence of ELC gradually increased. This trend existed among patients of different sexes, BMI, smoking, diabetes, and hypertension. To our knowledge, this study is the first to report the relationship between the occurrence of SAF‐AGEs and ELC.

ELC was first reported by Frank to be related to CHD, which is closely related to aging.^20^ ELC can be used to predict cardiovascular and cerebrovascular events^21^ and is related to the severity of coronary artery disease.^16^, ^20^ The prevalence of ELC increases with age and is mainly found in males.^16^ There is a significant association between ELC and myocardial histopathological changes.^6^ In the study of the Chinese population, the prevalence of ELC in patients without CHD confirmed by coronary angiography was 51.6%, whereas the prevalence of ELC in patients with CHD was 68.9%,^16^ demonstrating the predictive value of ELC for CHD in the Chinese population. Furthermore, there is a correlation between ELC findings and both hemorrhagic and ischemic stroke.^21^, ^22^ Consistent with previous studies, patients with ELC had higher rates of self‐reported CHD and stroke in the present study. However, no statistical difference in the occurrence of atrial fibrillation was found in patients with and without ELC.

The exact pathophysiology of ELC development is still unknown. Our study proposes that the formation of ELC is associated with the accumulation of AGEs. In previous studies, loss or degeneration of elastin fibers and breakage of elastic fibers connecting the earlobes due to the low oxygen saturation and microvascular disease may be potential mechanisms.^6^, ^23^ Telomeres of peripheral leukocytes were observed in a patient study involving Japanese men with ELC shortening, demonstrating that aging plays a role in the emergence of ELC.^24^ Due to the similarity of peptide chains on earlobe collagen and the macrophage receptor of scavengers of cholesterol intake, reduced macrophage receptor activity has also been hypothesized as a pathogenic mechanism for the development of ELC.^25^ According to Na et al, the occurrence of ELC was related to the polypeptide hormones adropin and irisin, which are associated with disorders of endothelial function.^26^ Abraham provided an anatomical explanation that the occurrence of ELC is related to long‐standing facial visceral obesity, especially the sideburn area of the cheek. As the deep cheek fat grows in patients with visceral obesity, the excess skin of the cheeks leads to skin pleating in the earlobes, which, together with the long‐term traction of the earlobe attachment, creates the anterior ear screen line and the anterior vertical crease of the ear, known as the ELC.^27^

T2DM is the key to aggravating macrovascular diseases such as atherosclerosis and microvascular diseases such as kidneys and fundus, and AGEs play an important role in diabetes‐related complications.^28^ In studies of diabetes and aging, accumulation of AGEs has been observed in joint collagen or the glomerular basement membrane.^29^, ^30^ One study showed that patients with ELC are at higher risk of developing blindness and diabetic retinopathy.^31^ Regarding the macrovascular complications of diabetes, AGEs contribute to the development of atherosclerosis through various mechanisms. AGEs modify proteins and extracellular matrices through glycation and cross‐linking, leading to structural changes. AGE accelerates the transformation of monocytes into macrophages by promoting the expression of endothelial cell adhesion molecules. Glycated LDL‐C is converted into to AGE‐modified LDL‐C, which is recognized by scavenger receptors, leading to the formation of foam cells.^32^ In addition, a previous study by our group identified the potential role of SAF‐AGEs in accelerating osteoporosis in diabetic patients.^18^ However, the role of T2DM in the development of ELC remains unclear. This study illustrates the correlation between the occurrence of ELC and the accumulation of AGE from a clinical perspective.

Compared to healthy controls, decreased skin elasticity was characterized in diabetic subjects.^33^ Collagen was identified as a scaffold for the mechanical support of cells and tissues. These structures provide tensile strength and elasticity, and the glycation of collagen has deleterious effects on biochemical properties, leading to stiffness and reduced flexibility.^11^ Glycated collagen resists degradation by matrix metalloproteinases, thereby inhibiting renewed functional collagen, ultimately leading to compromised tissue permeability and renewal.^34^ Studies have reported that elastin and fibronectin of the extracellular matrix are also damaged by AGEs, ultimately leading to dermal dysfunction.^35^ In Pageon's ex vivo study, ultraviolet irradiation and glycation increased skin inflammation and led to elastin deposition, which was involved in the degeneration of elastic tissue.^36^ Our study proposed a new perspective regarding the explanation of ELC formation, that is, the occurrence of ELC may be related to the accumulation of AGE.

According to the report, the prevalence rates of hypertension, atherosclerosis, and DM were found to be higher in patients with ELC as opposed to those without ELC. ELC patients are mainly male, significantly older, have higher rates of alcohol consumption, and have a higher prevalence of diabetes. The prevalence is higher among menopausal women compared with those who are premenopausal. However, there was no statistically significant difference in the prevalence of dyslipidemia, which is consistent with a previous report.^37^ The possible explanation is that this study was a community‐based population study, which may have interfered with the reported findings due to the fact that more participants in the ELC group applied lipid‐lowering medications. In addition, there may be errors based on self‐reported history of hyperlipidemia.

This study does, however, have certain limitations. First, the present study is a community‐based cross‐sectional study that lacks the correlation between ELC and prognosis of diseases such as cardiovascular; Secondy, CHD is a self‐reported prevalence and therefore differs from true CHD, which would substantially underestimate the prevalence of CHD. Third, age is a key factor affecting AGEs and ELC. However, the age of the study participants is relatively high and the conclusions may not be generalizable to the general population. Finally, the report only obtained the correlation between ELC and SAF‐AGEs and the speculation of the correlation from clinical analyses and lacked molecular biological validation, which may be the direction for future validation.

## 5 CONCLUSION

In conclusion, the research illustrated that ELC is associated with coronary heart disease, and offered a novel possibly clinical explanation, which is that the ELC occurrence is associated with SAF‐AGE, and SAF‐AGE may increase the risk of ELC occurrence.

## FUNDING INFORMATION

This research received no external funding.

## DISCLOSURE

The authors declare that they have no conflicts of interest.

## Data Availability

Further inquiries can be addressed to the corresponding author.
